# Myelin Changes in Poor Sleepers: Insights into Glymphatic Clearance Function and Regional Circadian Clock Gene Expression

**DOI:** 10.14336/AD.2024.0894

**Published:** 2025-08-13

**Authors:** Christina Andica, Koji Kamagata, Kaito Takabayashi, Zaimire Mahemuti, Manabu Iwasaki, Akifumi Hagiwara, Wataru Uchida, Hiroki Tabata, Hitoshi Naito, Hideyoshi Kaga, Yuki Someya, Yoshifumi Tamura, Ryuzo Kawamori, Hirotaka Watada, Shigeki Aoki

**Affiliations:** ^1^Department of Radiology, Juntendo University Graduate School of Medicine, Tokyo, Japan.; ^2^Faculty of Health Data Science, Juntendo University, Chiba, Japan.; ^3^Juntendo Advanced Research Institute for Health Science, Tokyo, Japan.; ^4^Department of Metabolism & Endocrinology, Juntendo University Graduate School of Medicine, Tokyo, Japan.; ^5^Sportology Center, Juntendo University Graduate School of Medicine, Tokyo, Japan.; ^6^Graduate School of Health and Sports Science, Juntendo University, Chiba, Japan.; ^7^Department of Sports Medicine and Sportology, Juntendo University Graduate School of Medicine, Bunkyo-ku, Japan.

**Keywords:** circadian clock genes, cognition, depression, glymphatic system, myelin, sleep

## Abstract

Sleep is essential for maintaining brain myelin integrity. Emerging evidence suggests that poor sleep quality compromises the glymphatic system, a perivascular network crucial for brain waste clearance, leading to the accumulation of neuroinflammatory and toxic proteins, which may affect myelin integrity. Furthermore, poor sleep quality results in alterations in gene expression within the brain. We evaluated the associations among poor sleep quality, brain myelin integrity, and glymphatic clearance function as well as the impact of circadian clock gene expression on regional cortical myelin content. 50 poor sleepers (average age 71.08 ± 4.69 years; Pittsburgh Sleep Quality Index [PSQI] > 5) and 50 good sleepers (average age 73.04 ± 5.80 years; PSQI ≤ 5) were assessed. Myelin volume fraction (MVF) was quantified using magnetization transfer saturation imaging, and glymphatic function was noninvasively examined using diffusion tensor imaging along the perivascular space. Circadian gene expression was analyzed using postmortem brain tissue from the Allen Human Brain Atlas. Magnetic resonance imaging measures were correlated with cognitive and depression scores. Lower MVF was observed in the fronto-temporo-parietal and limbic regions as well as in major white matter tracts in poor sleepers compared with that in good sleepers. This reduction was linked to lower cognitive function scores and higher depressive scores. Poor sleepers also exhibited lower diffusivity along the perivascular spaces, mediating the relationship between poor sleep quality and demyelination. Regions with higher expression of CLOCK, CRY2, PER1, and PER2 exhibited greater MVF disparities between good and poor sleepers, whereas lower expression of CRY1 was associated with more pronounced differences. Poor sleep quality was associated with lower brain myelin integrity, correlating with reduced cognitive performance and increased depressive symptoms. These changes might be mediated by glymphatic clearance dysfunction and were associated with the differential expression of circadian clock genes.

## INTRODUCTION

Sleep deprivation leads to brain myelin alterations [1]. Lower myelin content is correlated with rapid cognitive decline [2] and affective disorders [3]. Understanding the effects of poor sleep on myelin levels could enhance interventions aimed at brain health preservation.

Changes in diffusion tensor imaging (DTI) measures, including reduced fractional anisotropy (FA) and increased radial diffusivity (RD), which often indicate reduced myelin, were observed in the white matter (WM) of middle-aged and older adults with poor sleep quality [4, 5]. However, these results were not confirmed in larger [6] or younger cohorts [7]. FA and RD are affected by non-myelin factors that make them inadequate as measures of myelin [8].

Recent studies have explored alternative imaging modalities for assessing myelin content. The ratio of T1-weighted to T2-weighted images (T1w/T2w ratio) revealed a correlation between sleep deprivation and lower intracortical myelin content [7]. However, this ratio may reflect variations in axon density more than myelin content [9] and may not be suitable as a measure of myelin in WM [10]. A recent meta-analysis suggested that magnetization transfer (MT) imaging, which exploits the selective saturation of protons, such as those bound to myelin macromolecules [11], exhibits the highest correlation with histopathological myelin content [8]. In a recent study, MT imaging has been demonstrated to be suitable for the assessment of gray matter (GM) and WM myelin content [12], although further histopathological studies are warranted.

The glymphatic system, which facilitates brain waste clearance [13], is also affected by sleep quality [14]. The glymphatic system relies on aquaporin-4 channels in astrocytic endfeet to facilitate the influx of cerebrospinal fluid (CSF) into the brain interstitial space and the efflux of interstitial fluid (ISF) and metabolic waste to the perivenous spaces [13]. Sleep deprivation alters the expression of aquaporin-4 [15], impairing glymphatic clearance and leading to the accumulation of neuroinflammatory and neurotoxic substances, thereby resulting in myelin loss [16].

Magnetic resonance imaging (MRI) can visualize the human glymphatic system using gadolinium-based contrast agents administered intrathecally. However, because of the invasive nature and the risk of gadolinium deposition in brain tissues, its clinical use is limited. As an alternative, DTI along the perivascular space (ALPS) is a noninvasive and indirect method that assumes water diffusivity within the perivascular space correlates with ISF dynamics [17]. A significant association has been observed between the ALPS index and glymphatic clearance, as assessed using MRI following intrathecal gadolinium administration [18]. A lower ALPS index has been reported in individuals with poor sleep quality [19, 20].

Moreover, circadian clock genes exhibit sleep-related changes in the cerebral cortex [21]. These genes are suggested to regulate the proliferation of oligodendrocyte progenitor cells (OPCs) [22] and the expression of myelin proteolipid protein [23]. However, there is limited understanding of the mechanism by which the regional brain expression of circadian clock genes influences brain myelin content in the context of sleep disturbance.

We explored GM and WM myelin integrity in older adults with poor sleep quality via MT saturation (MTsat) imaging. To gain further insights into the mechanisms underlying sleep-related brain myelin changes, we determined whether reduced glymphatic system clearance, indicated by the ALPS index, could mediate demyelination. Finally, we evaluated the impact of circadian clock genes on the regional cortical myelin content.

## MATERIALS AND METHODS

### Study design and population

This cross-sectional study included older Japanese adults (age range, 65-82 years) who participated in the Bunkyo Health Study between March 2017 and September 2018 [24]. The study protocol was approved by the Ethics Committee of Juntendo University (first approval no. 2015078; most recent updated edition no. M15-0057-M08) and was conducted in accordance with the Declaration of Helsinki. All study participants provided written informed consent before participation.

**Figure 1.**
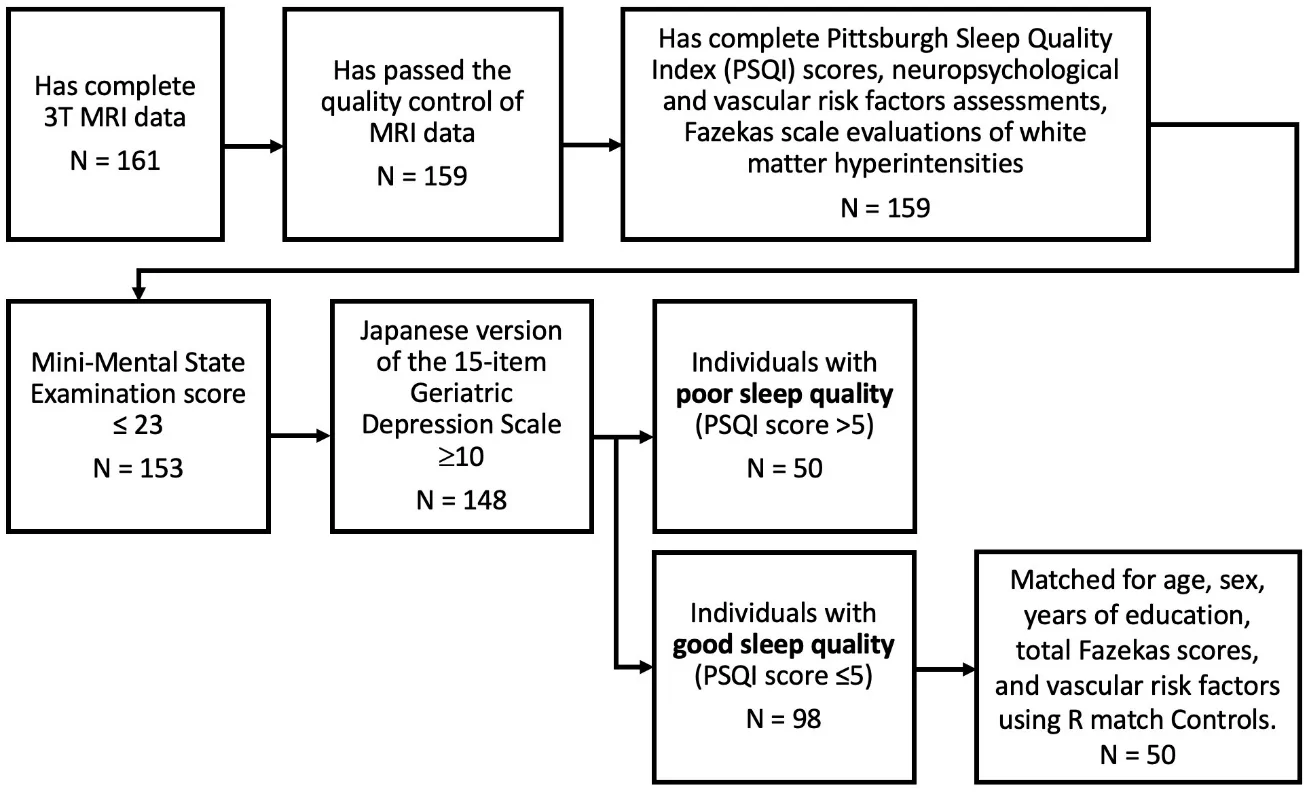
**Study participant flowchart**. This chart includes inclusion and exclusion criteria, along with the count of individuals meeting the criteria and included in the study population.

The inclusion and exclusion criteria of the study participants are shown in Figure 1. All study participants had no history of drug and/or alcohol abuse, past or present neurological or psychiatric disorders, or structural brain abnormalities observed on MRI.

### Sleep measures

In this study, the participants were categorized into good and poor sleeper groups based on their total Pittsburgh Sleep Quality Index (PSQI) scores. The PSQI is a self-reported questionnaire of sleep quality and disturbances over a 1-month time interval [25]. The PSQI includes seven subdimensions: 1. subjective sleep quality, 2. sleep latency, 3. sleep duration, 4. habitual sleep efficiency, 5. sleep disturbances, 6. use of sleeping medications and 7. daytime dysfunction, each weighted equally from 0 to 3. The PSQI is calculated by adding the scores of the seven subdimensions with a total score ranging from 0 to 21, where lower scores denote better sleep quality. Study participants with a total PSQI score of > 5 were categorized as ‘poor sleepers’ [25].

Furthermore, we matched good sleepers and poor sleepers in terms of age, sex, years of education, total Fazekas scores, and vascular risk factors, including body mass index, systolic and diastolic blood pressures, fasting plasma glucose, total cholesterol, high-density lipoprotein cholesterol, low-density lipoprotein cholesterol, triglycerides, and the cumulative Brinkman index of cigarette consumption (Fig. 1). Matching was performed using the matchControls package in R (www.rdocumentation.org/packages/e1071/versions/1.7-14/topics/matchControls).

### MRI acquisition

All MRI data were acquired using a 3-Tesla scanner (MAGNETOM Prisma; Siemens Healthcare, Erlangen, Germany) with a 64-channel head coil during daylight hours. The acquisition parameters of three-dimensional (3D) T1-weighted imaging, diffusion-weighted imaging, and MTsat imaging are provided in the supplementary materials.

### Myelin measurements

An in-house MATLAB script, based on a previous study [26], was used to calculate the myelin volume fraction (MVF) from the MTsat data. MVF denotes the fraction of a voxel occupied by myelin sheaths [27]. It is derived from linearly scaled MTsat maps [28] using a calibration factor of 0.1, as applied in several previous studies [29-31].

The MVF values in the regions of interest (ROIs) were obtained through skeletonized atlas-based segmentation for both GM and WM. Skeletonized segmentation reduced partial volume effects caused by misregistration across subjects. The skeletonization process involved the use of GM-based spatial statistics and tract-based spatial statistics for WM. The steps of the methods have been described in our previous studies [32, 33]. MVF values were then extracted across the ROIs superimposed onto the skeleton defined by the Desikan-Killiany atlas for GM and the JHU tractography atlas for WM. The Desikan-Killiany atlas comprises 34 cerebral cortices, the cerebellar cortex and seven subcortical regions in each hemisphere. Meanwhile, the JHU ICBM-DTI-81 WM tractography atlas contained 11 WM tracts in each hemisphere. The average MVF values from both hemispheres were then used in the subsequent statistical analyses.

### Calculation of the ALPS index

**Figure 2.**
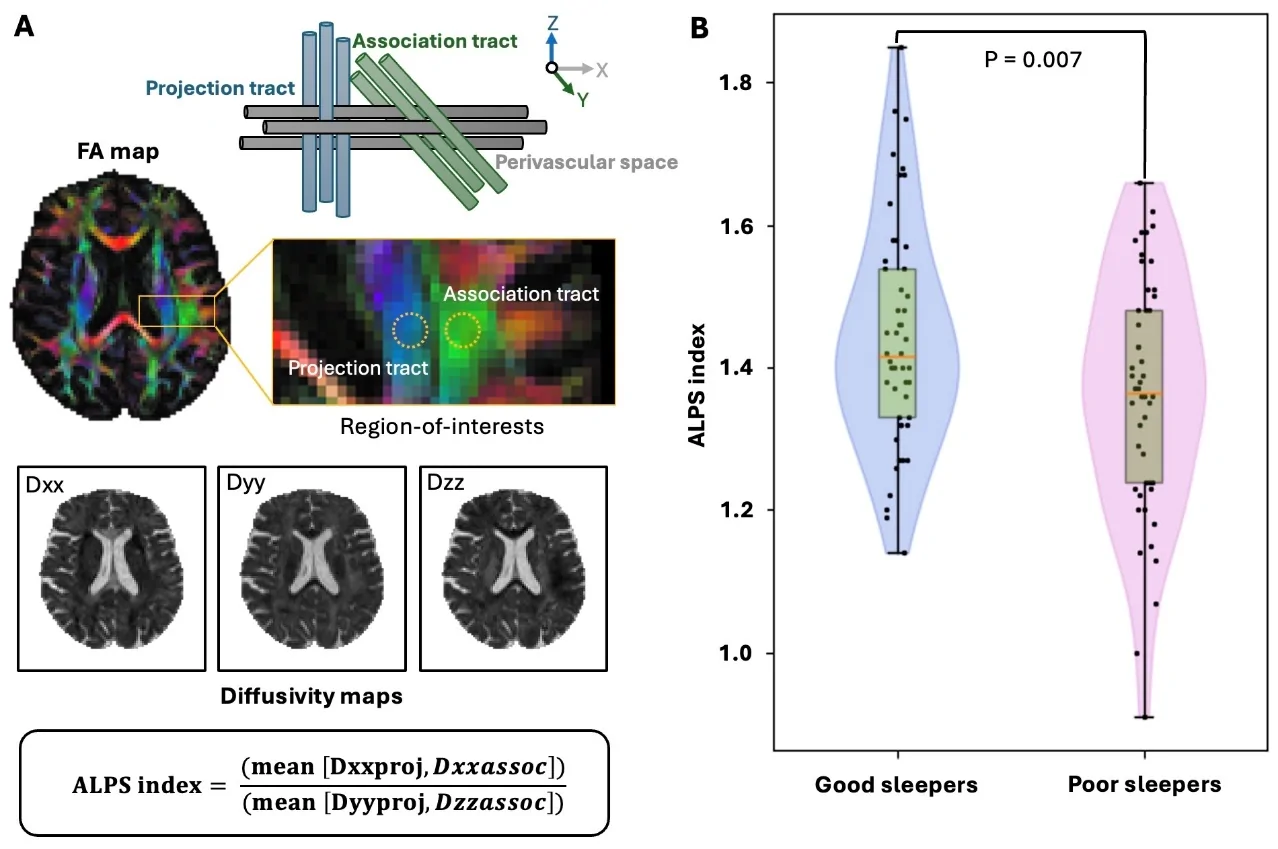
**Assessment of diffusion tensor imaging index along the perivascular space (ALPS index)**. (**A**) Calculation of the ALPS index. Color-coded fractional anisotropy (FA) map showing the distribution of the projection (blue, z-axis) and association (green, y-axis) fibers at the lateral ventricle level of the body. Spherical regions of interest measuring 5 mm in diameter were positioned in the areas of the projection and association fibers. The perivascular space runs perpendicular to the projection and association tracts. Diffusivity maps in the x-axis (Dxx), y-axis (Dyy) and z-axis (Dzz). The ALPS index was derived from the ratio of the mean x-axis diffusivity in the projection area (Dxxproj) and x-axis diffusivity in the association area (Dxxassoc). (**B**) The ALPS index in groups without and with sleep disturbances. The *P*-value corresponds to a univariate general linear model after controlling for age, sex, years of education, intracranial volume, mean arterial pressure, smoking habit, and FA value.

The ALPS index was calculated using a validated semiautomated pipeline [34]. Initially, the FA maps of all study participants were registered into the FMRIB58_FA standard space using both linear and nonlinear transformations. For the placement of ROIs, the subject with the least degree of warping was selected. Using this subject’s color-coded FA map, spherical ROIs measuring 5 mm in diameter were placed in the projection and association areas at the level of the bilateral lateral ventricle body (Fig. 2A). The resulting ROIs were then registered to the same FA template, and their position was visually confirmed for each participant. Manual adjustments were performed to the ROI placements when necessary.

In the area of the projection fibers, the primary fibers are oriented along the z-axis, with the x- and y-axes being perpendicular to these main fibers. Conversely, in the area of the association fibers, the primary fibers are aligned with the y-axis, whereas the x- and z-axes lie perpendicular to the main fibers. Thus, the ALPS index was calculated from the ratio of the average values of the x-axis diffusivity in the area of the projection fibers (Dxxproj) and the x-axis diffusivity in the area of the association fibers (Dxxassoc) to the average values of the y-axis diffusivity in the area of the projection fibers (Dyyproj) and the z-axis diffusivity in the area of the association fibers (Dzzassoc), as follows:

$$\mathrm{ALPS index}=\frac{\left(\mathrm{mean}\left[\mathrm{Dxxproj},\mathrm{Dxxassoc}\right]\right)}{\left(\mathrm{mean}\left[\mathrm{Dyyproj},\mathrm{Dzzassoc}\right]\right)}$$

The ALPS index is the ratio of diffusivity parallel to the perivascular spaces around the deep medullary veins to that perpendicular to the WM fiber tracts at the level of the lateral ventricles, noninvasively inferring glymphatic activities in the human brain. The average values of the left and right ALPS indices were evaluated in this study. An ALPS index closes to 1 reflects minimal diffusion along the perivascular space, whereas a larger ratio indicates larger water diffusivity along the perivascular space [17].

The ALPS index does not solely quantify the diffusivity of the perivenous spaces around the deep medullary veins; it is also affected by the WM microstructure within the ROIs. Therefore, we also obtained FA values using the same ROIs, which are indicative of WM integrity in the association and projection fibers. Statistical analyses then incorporated the FA values from the projection and association fibers, ensuring that the ALPS index reflects the diffusivity of the perivenous spaces rather than WM integrity [35, 36].

### Circadian clock regional gene expression

Data on the gene expressions of circadian locomotor output cycles kaput (CLOCK), cryptochrome (CRY) 1 and 2, and period (PER) 1, 2, and 3 were obtained from the Allen Human Brain Atlas (AHBA; https://human.brain-map.org). The AHBA provides a comprehensive view of the gene expression profile across the human brain [37]. These data were used to investigate the association between regional patterns of MVF differences between groups and gene expression. In this study, we used the Abagen toolbox (https://abagen.readthedocs.io) to extract regional gene expression based on the Desikan-Killiany atlas using default settings [37-39]. Considering that the AHBA provides right hemisphere data for only two subjects, we restricted our analysis to values from the left hemisphere [39-41]. Before averaging across all individuals, the gene expression values were normalized within each of the six individual donor brains using the scaled robust sigmoid normalization method, which is included in the default settings of the Abagen toolbox. The normalization step was performed to adjust for potential intersample differences and donor-specific effects in the transcriptome [42].

### Brain volume measurements

Intracranial (ICV) volume was obtained from each study participant using T1-weighted images in FreeSurfer version 6.0.0 (http://surfer.nmr.mgh.harvard.edu/fswiki) with the recon-all pipeline [43]. The resulting segmentations were visually checked, and manual adjustments were made as necessary. The ICV was used as a nuisance covariate in the statistical analyses.

### Statistical analysis

All statistical analyses were performed using Statistical Package for the Social Sciences (Macintosh version 27; Armonk, NY, IBM Corporation). The normality of the data was assessed using the Kolmogorov-Smirnov test. Demographic and clinical data of good and poor sleepers (Table 1) were analyzed using the independent-samples *t*-tests or Mann-Whitney *U* tests for normally or nonnormally distributed continuous data, respectively, whereas the chi-squared test was used for categorical variables. *P*-values of <0.05 were used to denote statistical significance.

The ALPS index and GM and WM MVF were compared between good and poor sleepers using a general linear model, while controlling for potential covariates, including age, sex, years of education, ICV, mean arterial pressure (MAP) (MAP = diastolic + 1/3 [systolic - diastolic]) and smoking habit (yes [Brinkman index ≥ 1]/no [Brinkman index = 0]). All of these factors have been demonstrated to be associated with myelin content and the glymphatic system [35, 44, 45]. Furthermore, FA values were included as covariates in the assessment of the ALPS index. Multiple comparisons of MVF in the WM tracts (11 ROIs) and GM areas (42 ROIs) were adjusted using the false discovery rate (FDR). FDR-corrected *P*-values of < 0.05 were used to denote statistical significance.

Multiple linear regression (MLR) analyses were performed to model the associations between MRI-based measures (independent variables) and PSQI scores or neurophysiological scores (dependent variables), including Mini-Mental State Examination (MMSE), the Japanese version of the Montreal Cognitive Assessment (MoCA-J), Trail Making Test (TMT) B-A and Japanese version of the 15-item Geriatric Depression Scale (GDS-15-J) scores. In the evaluation of the ALPS index, covariates such as group (good or poor sleepers), age, sex, years of education, ICV, MAP, smoking habit, and FA values were included. The MLR analysis was performed only on GM and WM ROIs exhibiting significant differences in MVF between good and poor sleepers, while controlling for group (good or poor sleepers), age, sex, years of education, ICV, MAP, and smoking habits. Backward elimination was used for variable selection. Multicollinearity of predictor variables was addressed by assessing variance inflation factor (VIF) values, with VIF values ≤ 10 indicating the absence of multicollinearity. *P*-values (two-tailed) of < 0.05 were used to denote statistical significance.

**Figure 3.**
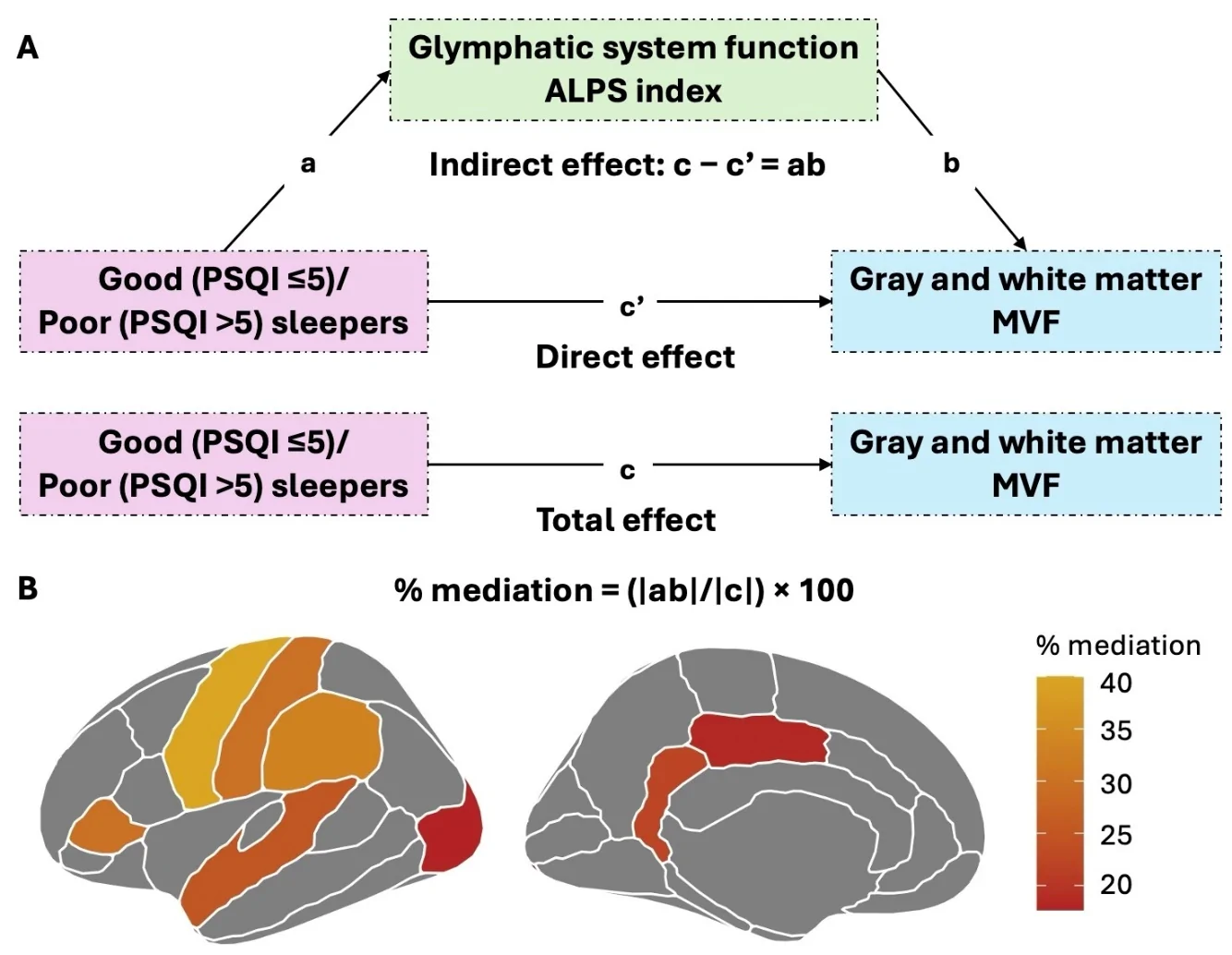
**Mediation analysis between the ALPS index and MVF**. (**A**) Schema of mediation analysis between sleep disturbance, glymphatic system function measured using the ALPS index and gray and white matter MVF. (**B**) Gray matter regions with the ALPS index as a significant mediator of the relationship between poor sleep quality and gray matter MVF. Significant regions are plotted based on the percent mediation. Nonsignificant regions are in gray. Abbreviations: ALPS, analysis along the perivascular space; MVF, myelin volume fraction; PM, percent mediation; PSQI, Pittsburgh Sleep Quality Index.

To assess the role of the ALPS index as a mediator between sleep disturbance and MVF values of GM and WM ROIs, we performed mediation analysis using the PROCESS macro for Statistical Package for the Social Sciences (model 4). Mediation analysis is a statistical approach used to quantify a mediating variable in the causal sequence by which an antecedent variable is caused by a dependent variable [46]. The mediation analysis encompassed the total, direct, and indirect effects, as illustrated in Figure 3A. This analysis was performed with a confidence level set at 95% and 5,000 bootstrap samples. The covariates included in the analysis were age, sex, years of education, ICV, MAP, smoking habit, and FA values. The analysis was performed only on GM and WM ROIs exhibiting significant differences in MVF between good and poor sleepers. The percent mediation, calculated by dividing the indirect effect by the total effect, was then used to examine the weight of the ALPS index in the total effect. Statistical significance was defined as *P*-values of < 0.05, and the indirect effect was considered significant if the confidence intervals did not encompass zero.

The associations between the regional expression of CLOCK, CRY1, CRY2, PER1, PER2 and PER3 and differences in GM MVF between good and poor sleepers in 41 ROIs (excluding the cerebellar cortex) of the Desikan-Killiany atlas were tested using partial correlation analysis, accounting for age, sex, years of education, ICV, MAP, and smoking habit as covariates. For this analysis, the MVF values in good and poor sleepers were separately averaged across 41 ROIs, and the absolute differences between the groups were then calculated.

## RESULTS

### Study participants’ demographic and clinical characteristics

In this study, we analyzed 100 subjects aged 65-84 years, including 50 good sleepers (24 men and 26 women; mean age, 71.08 ± 4.69 years) and 50 poor sleepers (24 men and 26 women; mean age, 73.04 ± 5.80 years), who were matched for age, sex, years of education, total Fazekas score, and vascular risk factors. The demographic and clinical characteristics of the participants are summarized in Table 1.

**Table 1:** Demographic characteristics of the study participants without and with sleep disturbances.

	Without	With	*P*-value
**No. of participants**	50	50	
**Sex (N; male/female)**	24/26	24/26	1.00
**Age (years)**	71.08 ± 4.69	73.04 ± 5.80	0.093
**Education (years)**	14.46 ± 1.83	14.06 ± 1.97	0.313
**PSQI:**			
**1. Sleep quality (N; 0/1/2/3)**	11/37/2/0	2/23/23/2	<0.001
**2. Sleep-onset latency (N; 0/1/2/3)**	33/17/0/0	2/14/9/34	<0.001
**3. Sleep duration (N; 0/1/2/3)**	10/24/15/1	8/10/26/6	0.006
**4. Sleep efficiency (N; 0/1/2/3)**	44/6/0/0/	28/11/4/7	0.001
**5. Sleep disturbance (N; 0/1/2/3)**	15/35/0	1/47/2	<0.001
**6. Use of sleep medication (N; 0/1/2/3)**	47/2/1/0	32/3/4/11	0.001
**7. Daytime dysfunction (N; 0/1/2)**	34/14/2	31/15/4	0.657
**Total score**	3.56 ± 0.56	8.58 ± 2.35	<0.001
**Vascular risk factors:**			
**Body mass index (kg/m^2^)**	22.74 ± 2.08	23.20 ± 2.81	0.352
**Systolic blood pressure (mmHg)**	134.14 ± 13.88	137.98 ± 19.04	0.252
**Diastolic blood pressure (mmHg)**	84.22 ± 8.42	86.92 ± 8.81	0.121
**Mean arterial pressure (mmHg)**	100.86 ± 9.28	103.94 ± 11.26	0.139
**Total cholesterol (mg/dL)**	205.12 ± 29.38	211.54 ± 54.31	0.890
**HDL cholesterol (mg/dL)**	66.18 ± 13.13	62.18 ± 15.99	0.175
**LDL cholesterol (mg/dL)**	119.52 ± 28.07	124.22 ± 32.25	0.439
**Triglycerides (mg/dL)**	95.24 ± 51.13	105.36 ± 77.24	0.770
**Fasting plasma glucose (mg/dL)**	95.78 ± 8.17	97.04 ± 8.82	0.302
**HbA1c (mmol/mol)**	5.64 ± 0.31	5.72 ± 0.38	0.179
**Smoking habit, (N; No/Yes)**	30/20	27/23	0.545
**Brinkman index**	282.62 ± 440.56	301.25 ± 534.38	0.691
**Neuropsychological measures:**			
**MMSE**	28.22 ± 1.54	27.92 ± 1.51	0.273
**MoCA-J**	25.88 ± 2.62	24.86 ± 3.24	0.158
**TMT B-A**	60.10 ± 29.22	77.24 ± 50.16	0.070
**GDS-15-J**	1.86 ± 1.80	2.60 ± 2.17	0.085
**Fazekas scale:**			
**Periventricular white matter (N; 0/1/2/3)**	0/45/5/0	1/39/10/0	0.213
**Deep white matter (N; 0/1/2/3)**	0/38/12/0	1/32/14/3	0.198
**Total Fazekas scale**	2.34 ± 0.63	2.56 ± 0.93	0.223
**Other measures:**			
**Intracranial volume (mL)**	1411.29 ± 145.59	1392.76 ± 134.61	0.510
**Mean fractional anisotropy**	0.54 ± 0.03	0.53 ± 0.04	0.654

Abbreviations: GDS-15-J, 15-item Geriatric Depression Scale; HDL, high-density lipoprotein; LDL, low-density lipoprotein; MMSE, Mini-Mental State Examination; MoCA-J, Japanese version of the Montreal Cognitive Assessment; PSQI, Pittsburgh Sleep Quality Index; TMT, Trail Making Test.

No significant differences in terms of age, sex, years of education, vascular risk factors, the Fazekas scale, MMSE, MoCA-J, TMT B-A, and GDS-15-J scores were observed between good and poor sleepers.

Poor sleepers had significantly higher total PSQI scores and subscores (i.e. subjective sleep quality, sleep latency, sleep duration, habitual sleep efficiency, sleep disturbances, and use of sleeping medication). However, the PSQI subscore of daytime dysfunctions was not significantly different between good and poor sleepers.

### Differences in GM and WM MVF and the ALPS index between the groups

We observed significantly lower (FDR-corrected *P* < 0.05) MVF in various GM regions, including the frontal lobe (i.e. caudal middle frontal, lateral and medial orbitofrontal, pars triangularis, precentral and superior frontal), temporal lobe (i.e. bankssts, inferior, middle and superior temporal), parietal lobe (i.e. inferior parietal, postcentral, precuneus and supramarginal), occipital lobe (i.e. fusiform and lateral occipital), limbic areas (i.e. caudal anterior, isthmus, posterior and rostral anterior cingulate and insula) and deep GM regions (i.e. caudate, putamen, hippocampus and thalamus) in poor sleepers than in good sleepers (Supplementary Table 1). Furthermore, poor sleepers also exhibited significantly lower MVF than good sleepers in the WM tracts, including anterior thalamic radiation (ATR), corticospinal tract (CST), cingulum, cingulum hippocampus, forceps major, forceps minor, inferior fronto-occipital fasciculus (IFOF), inferior longitudinal fasciculus (ILF), superior longitudinal fasciculus (SLF) and uncinate fasciculus (UF) (Supplementary Table 2).

Poor sleepers had a significantly lower ALPS index (1.36 ± 0.17; *p* = 0.007) than good sleepers (1.44 ± 0.16) (Fig. 2B). Furthermore, no significant differences in FA values measured in the same ROIs used to obtain the ALPS index were observed between the two groups (Table 1).

### Associations between MRI-based measures and PSQI or neuropsychological scores

#### GM MVF

In the MLR analyses, lower MVF values in the following regions were associated with higher PSQI scores:

PSQI 1 score: inferior temporal (standardized β, -0.77 [standard error {SE}: 5.26]; *P* < 0.001; VIF = 5.03) and caudate (standardized β, -0.35 [SE: 3.14]; *P* < 0.001; VIF = 1.69).

PSQI 2 score: lateral orbitofrontal (standardized β, -0.38 [SE: 6.74]; *P* < 0.001; VIF = 3.93) and inferior parietal (standardized β, -0.28 [SE: 5.73]; *P* = 0.005; VIF = 3.40).

PSQI 3 score: pars triangularis (standardized β, -0.39 [SE: 5.57]; *P* = 0.010; VIF = 2.70), inferior temporal (standardized β, -0.40 [SE: 9.62]; *P* = 0.043; VIF = 4.76) and superior temporal (standardized β, -0.47 [SE: 9.60]; *P* = 0.028; VIF = 5.40).

PSQI 4 score: supramarginal (standardized β, -0.43 [SE: 6.20]; *P* = 0.005; VIF = 2.83) and isthmus cingulate (standardized β, -0.29 [SE: 6.84]; *p* = 0.043; VIF = 2.63).

PSQI 5 score: middle temporal (standardized β, -0.34 [SE: 2.99]; *P* = 0.013; VIF = 2.29).

PSQI 6 score: caudal middle frontal (standardized β, -0.37 [SE: 4.39]; *P* = 0.007; VIF = 2.58).

PSQI 7 score: caudal middle frontal (standardized β, -0.39 [SE: 2.82]; *P* = 0.009; VIF = 2.51) and rostral anterior cingulate (standardized β, -0.55 [SE: 5.03]; *P* < 0.001; VIF = 3.04).

PSQI total score: caudal middle frontal (standardized β, -0.32 [SE: 8.42]; *P* < 0.001; VIF = 2.82), inferior parietal (standardized β, -0.21 [SE: 12.90]; *P* = 0.023; VIF = 3.18) and supramarginal (standardized β, -0.21 [SE: 13.81]; *P* = 0.29; VIF = 3.36).

Furthermore, lower MVF values in the following regions were associated with lower MMSE and MoCA-J scores and higher GDS-15-J scores:

MMSE: caudal middle frontal (standardized β, 0.33 [SE: 6.54]; *P* = 0.015; VIF = 2.59), superior temporal (standardized β, 0.50 [SE: 17.08]; *P* = 0.021; VIF = 6.41), supramarginal (standardized β, 0.31 [SE: 11.18]; *p* = 0.045; VIF = 3.37), isthmus cingulate (standardized β, 0.31 [SE: 12.67]; *p* = 0.043; VIF = 3.31) and putamen (standardized β, 0.33 [SE: 16.65]; *P* = 0.043; VIF = 3.78).

MoCA-J: thalamus (standardized β, 0.27 [SE: 28.40]; *P* = 0.030; VIF = 1.97).

GDS-15-J: lateral occipital (standardized β, -0.37 [SE: 9.38]; *P* = 0.002; VIF = 1.79) and rostral anterior cingulate (standardized β, -0.54 [SE: 18.33]; *P* = 0.003; VIF = 4.02).

#### WM MVF

In the MLR analyses, lower MVF values in the following regions were associated with higher PSQI scores:

PSQI 3 score: ILF (standardized β, -0.33 [SE: 5.20]; *P* = 0.019; VIF = 2.20).

PSQI 4 score: forceps minor (standardized β, -0.33 [SE: 2.14]; *P* = 0.041; VIF = 3.23).

PSQI 5 score: SLF (standardized β, -0.20 [SE: 3.81]; *P* = 0.034; VIF = 1.09).

Furthermore, lower MVF values in the following WM tracts were associated with lower MMSE and higher TMT B-A scores:

MMSE score: ILF (standardized β, 0.50 [SE: 13.49]; *P* = 0.016; VIF = 5.37).

TMT B-A score: IFOF (standardized β, -0.72 [SE: 358.35]; *P* < 0.001; VIF = 5.86).

#### ALPS index

No significant associations were observed between the ALPS index and PSQI or neuropsychological scores.

### The ALPS index as a mediator between poor sleep quality and GM and WM demyelination

In the mediation analysis, glymphatic system clearance, as measured using the ALPS index, demonstrated significant mediation effects between sleep quality and GM (Fig. 3B and Table 2) and WM (Table 3) MVF. In particular, the ALPS index fully mediated the association between poor sleep quality and GM demyelination in the pars triangularis, precentral, superior temporal, postcentral, and supramarginal regions. Furthermore, it served as a partial mediator in the lateral occipital, isthmus, and posterior cingulate areas. Meanwhile, the ALPS index fully mediated the association between poor sleep quality and WM demyelination in the CST, cingulum, and SLF. Furthermore, it also acted as a partial mediator in the forceps major.

**Table 2:** Mediation analyses among sleep quality, ALPS index, and gray matter MVF.

Gray matter areas	a		b		Direct effect (c’)		Indirect effect			Total effect (c)	Percentage mediation
	Coefficient	*P*-values	Coefficient	*P*-values	Effect	*P*-values	Effect	LLCI	ULCI		
**Frontal lobe**											
**Caudal middle frontal**	-0.084	0.006	0.030	0.130	-0.016	0.008	-0.003	-0.008	0.001	-0.019	15.79
**Lateral orbitofrontal**	-0.084	0.006	0.015	0.210	-0.007	0.042	-0.001	-0.004	0.0003	-0.008	12.50
**Medial orbitofrontal**	-0.084	0.006	0.016	0.210	-0.010	0.008	-0.001	-0.004	0.0006	-0.011	9.09
**Pars triangularis**	-0.084	0.006	0.031	0.030	-0.007	0.077	-0.003	-0.006	-0.0001	-0.010	30.00
**Precentral**	-0.084	0.006	0.046	0.001	-0.006	0.130	-0.004	-0.009	-0.0005	-0.010	40.00
**Superior frontal**	-0.084	0.006	0.039	0.018	-0.012	0.017	-0.003	-0.009	0.0003	-0.015	20.00
**Temporal lobe**											
**Bankssts**	-0.084	0.006	0.017	0.160	-0.009	0.010	-0.001	-0.004	0.0004	-0.010	10.00
**Fusiform**	-0.084	0.006	0.012	0.210	-0.007	0.014	-0.001	-0.004	0.0008	-0.008	12.50
**Inferior temporal**	-0.084	0.006	0.006	0.570	-0.007	0.022	-0.0005	-0.003	0.001	-0.008	6.67
**Middle temporal**	-0.084	0.006	0.019	0.100	-0.008	0.033	-0.002	-0.005	0.0004	-0.010	20.00
**Superior temporal**	-0.084	0.006	0.028	0.016	-0.006	0.076	-0.002	-0.006	-0.0003	-0.008	25.00
**Parietal lobe**											
**Inferior parietal**	-0.084	0.006	0.017	0.200	-0.008	0.035	-0.001	-0.005	0.001	-0.009	11.11
**Postcentral**	-0.084	0.006	0.039	0.002	-0.007	0.077	-0.003	-0.007	-0.0006	-0.010	30.00
**Precuneus**	-0.084	0.006	0.024	0.028	-0.007	0.036	-0.002	-0.005	0.0005	-0.009	22.22
**Supramarginal**	-0.084	0.006	0.031	0.017	-0.006	0.120	-0.003	-0.006	-0.0002	-0.009	33.33
**Occipital lobe**											
**Lateral occipital**	-0.084	0.006	0.035	0.021	-0.014	0.002	-0.003	-0.006	-0.0003	-0.017	17.65
**Limbic regions**											
**Caudal anterior cingulate**	-0.084	0.006	0.028	0.032	-0.010	0.014	-0.002	-0.006	0.0001	-0.012	16.67
**Isthmus cingulate**	-0.084	0.006	0.025	0.014	-0.007	0.015	-0.002	-0.005	-0.0004	-0.009	22.22
**Posterior cingulate**	-0.084	0.006	0.024	0.035	-0.009	0.006	-0.002	-0.005	-0.0002	-0.011	18.18
**Rostral anterior cingulate**	-0.084	0.006	0.015	0.210	-0.010	0.005	-0.001	-0.004	0.0006	-0.011	9.09
**Insula**	-0.084	0.006	0.011	0.250	-0.007	0.015	-0.0009	-0.003	0.0005	-0.0079	11.39
**Deep gray matter**											
**Thalamus**	-0.084	0.006	0.010	0.200	-0.007	0.006	-0.0008	-0.002	0.0004	-0.0078	10.26
**Caudate**	-0.084	0.006	0.006	0.690	-0.009	0.040	-0.0005	-0.003	0.002	-0.0095	5.26
**Putamen**	-0.084	0.006	0.005	0.640	-0.008	0.007	-0.0004	-0.002	0.0009	-0.0084	4.76
**Hippocampus**	-0.084	0.006	-0.005	0.530	-0.006	0.018	0.0004	-0.0007	0.002	-0.0056	-7.14

Bold values denote statistical significance. Abbreviations: ALPS, along the perivascular space; LLCI, lower limit confidence interval; MVF, myelin volume fraction; ULCI, upper limit confidence interval.

### Regional association between GM MVF and CLOCK, CRY1, CRY2, PER1, PER2, and PER3 regional gene expression

**Figure 4.**
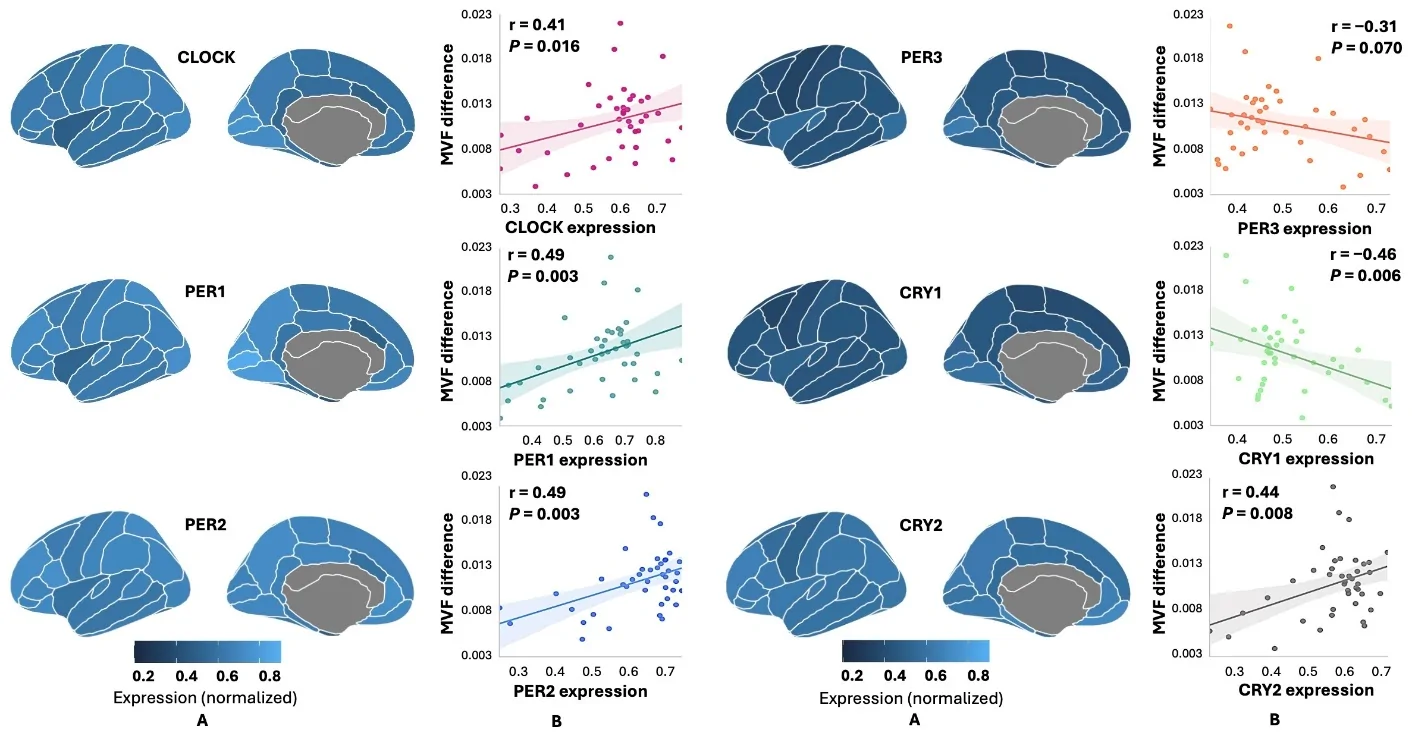
**Association between the brain expression of CLOCK, CRY1, CRY2, PER1, PER2 and PER3 genes and differences in gray matter myelin volume fraction (MVF) between good and poor sleepers**. (**A**) Distribution of CLOCK, CRY1, CRY2, PER1, PER2 and PER3 gene expression across gray matter regions of the left hemisphere analyzed using postmortem tissue provided by the Allen Human Brain Atlas. Before averaging across all individuals, values were normalized within each of the six individual donor brains. (**B**) Scatter plots of the correlation between CLOCK, CRY1, CRY2, PER1, PER2, or PER3 gene expression and group differences in MVF, while controlling for age, sex, years of education, ICV, MAP, and smoking habit. Each data point denotes the absolute differences in the MVF values between good and poor sleepers across 41 gray matter regions of interest. The MVF values in each region were first averaged separately within the good and poor sleeper groups, and then the differences between these group averages were calculated.

We observed significant positive correlations between MVF differences and the gene expression of CLOCK (r = 0.41, *P* = 0.016), PER1 (r = 0.49, *P* = 0.003), PER2 (r = 0.49, *P* = 0.003) and CRY2 (r = 0.44, *P* = 0.008) in the GM areas (Fig. 4). In contrast, a significant negative correlation was observed between MVF differences and CRY1 gene expression (r = -0.46, *P* = 0.006) (Fig. 4). No significant correlation was observed between MVF differences and PER3 gene expression (r = -0.31, *P* = 0.070).

## DISCUSSION

This study explored the role of glymphatic clearance in the relationship between sleep quality and myelination in GM and WM. Furthermore, we investigated the associations between the regional expression of circadian clock genes and changes in cortical myelin content. Key findings include the following: (1) poor sleepers demonstrated reduced GM and WM myelination and lower diffusivity along the perivascular spaces, which might indicate reduced glymphatic clearance function, compared with good sleepers; (2) reduced glymphatic system function was identified as a mediator in the demyelination of both GM and WM associated with poor sleep; and (3) spatial differences in GM MVF between good and poor sleepers were significantly correlated with regional variations in the expression of circadian clock genes.

**Table 3:** Mediation analyses among sleep quality, ALPS index, and white matter tract MVF.

White matter tracts	a		b		Direct effect (c’)		Indirect effect			Total effect (c)	Percentage mediation
	Coefficient	*P*-values	Coefficient	*P*-values	Effect	*P*-values	Effect	LLCI	ULCI		
**ATR**	-0.084	0.006	0.031	0.043	-0.011	0.013	-0.003	-0.006	0.000	-0.014	19.12
**CST**	-0.084	0.006	0.041	0.008	-0.006	0.200	-0.004	-0.007	-0.0008	-0.010	36.84
**Cingulum**	-0.084	0.006	0.058	0.001	-0.008	0.130	-0.005	-0.010	-0.001	-0.013	37.98
**Cingulum hippocampus**	-0.084	0.006	0.034	0.081	-0.011	0.053	-0.003	-0.009	0.002	-0.014	20.86
**Forceps major**	-0.084	0.006	0.041	0.006	-0.009	0.044	-0.003	-0.008	-0.0001	-0.012	27.42
**Forceps minor**	-0.084	0.006	0.024	0.220	-0.017	0.004	-0.002	-0.007	0.002	-0.019	10.53
**IFOF**	-0.084	0.006	0.032	0.034	-0.012	0.013	-0.003	-0.007	0.0001	-0.015	18.37
**ILF**	-0.084	0.006	0.032	0.027	-0.011	0.014	-0.003	-0.007	0.0001	-0.014	19.71
**SLF**	-0.084	0.006	0.061	<0.001	-0.007	0.130	-0.005	-0.011	-0.001	-0.012	42.15
**UF**	-0.084	0.006	0.020	0.170	-0.012	0.008	-0.002	-0.005	0.0005	-0.014	12.41

Bold values denote statistical significance. Abbreviations: ALPS, along the perivascular space; ATR, anterior thalamic radiation; CST, corticospinal tract; IFOF, inferior fronto-occipital fasciculus; ILF, inferior longitudinal fasciculus; LLCI, lower limit confidence interval; MVF, myelin volume fraction; SLF, superior longitudinal fasciculus, UF, uncinate fasciculus; ULCI, upper limit confidence interval.

### Associations between poor sleep quality and myelin alterations in GM and WM

We observed significantly reduced MVF values across extensive GM regions and major WM tracts in older adults with poor sleep quality compared with those with good sleep patterns, even after correcting for multiple comparisons and adjusting for multiple covariates. Prolonged wakefulness might compromise myelin integrity by affecting oligodendrocytes. Because oligodendrocytes require substantial energy, the energy used to support neurons during an extended period of wakefulness may result in myelin thinning [47].

Differences in MVF within the GM were predominantly observed in the fronto-temporo-parietal and limbic regions, including regions of the default mode network (DMN). Lower MVF values were significantly associated with the total score and subscores in the PSQI in overlap regions. Functional connectivity changes were observed in the fronto-temporo-parietal and limbic brain areas in poor sleepers, consistent with the findings of a previous study [48]. Furthermore, reduced functional connectivity of the DMN during both rest and task performance was associated with sleep deprivation in older adults [49].

Moreover, our findings suggest that reductions in myelin within the caudal middle frontal, superior temporal, supramarginal, isthmus cingulate, putamen, and thalamus are associated with global cognitive decline. These brain regions contribute to the complex network that supports cognitive function and is involved in the pathology of Alzheimer’s disease [50]. Lower MVF values were also associated with depressive symptoms, particularly in the lateral occipital and rostral anterior cingulate regions. The malfunctioning rostral anterior cingulate cortex, which is a highly interconnected hub within the DMN, along with the lateral occipital cortex, which is part of the visual cortex, is consistently believed to contribute to the onset, maintenance and recurrence of depression [51].

Reductions in MVF values were also observed in major WM tracts, including the ATR, CST, cingulum, forceps major and minor, IFOF, ILF, SLF, and UF, which have previously been shown to be involved in sleep disorders [4, 5, 52]. Interestingly, in line with a previous study [53], larger effect sizes were observed in associative fibers (i.e. IFOF, ILF and UF), ATR, forceps minor, cingulum, and CST. Associative fibers, ATR, and forceps minor have been shown to have a stronger correlation with aging, whereas other white matter tracts exhibited modest or no correlation [53]. Considering that aging is metabolically associated with and significantly influences sleep quality [54], these WM tracts may be more vulnerable to the effects of sleep deprivation. This hypothesis is supported by the observation that PSQI subscores were significantly correlated only with the forceps minor, ILF, and SLF.

Furthermore, lower MVF values in the ILF and IFOF were associated with declines in global cognitive function, as measured using the MMSE score and executive function, as assessed using the TMT B-A score. The ILF, which serves as the primary associative fiber tract, connects the occipital and ventro-anterior temporal lobes. Compromised integrity of the ILF is associated with various neuropsychological issues, including thought disorders, disturbances in processing visual emotions, cognitive deficits such as impaired semantic fluency and mood disorders [55]. In contrast, the IFOF, which is known as the longest associative bundle in the human brain, facilitates connections between various parts of the occipital cortex, temporobasal area, and superior parietal lobule with the frontal lobe. Deficits in executive function have been associated with alterations in the WM structures connecting to the frontal lobes [56].

### Dysfunctions in glymphatic clearance in poor sleepers and their correlation with GM and WM demyelination

Consistent with prior research [19, 20], our poor sleep quality group exhibited significantly lower ALPS indices than those with good sleep quality. A lower ALPS index suggests lower diffusivity along the perivascular spaces, which might indicate impaired CSF-ISF drainage toward the perivenous space, potentially due to dysfunction in glymphatic clearance due to poor sleep [17]. In support of our findings, a previous study demonstrated that sleep deprivation impairs the clearance of gadolinium contrast agent administered intrathecally from most brain regions of healthy individuals [57].

Furthermore, a mediation analysis revealed that a lower ALPS index mediates demyelination in several GM and WM regions associated with poor sleep quality. Sleep deprivation has been demonstrated to increase the CSF levels of amyloid-β, tau, and α-synuclein [58, 59] and to be associated with microglial morphological changes and increased expression of pro-inflammatory cytokines [60]. Studies have indicated that the accumulation of amyloid-β, tau, α-synuclein and inflammatory substances due to stagnating glymphatic clearance could contribute to myelin breakdown, leading to the progressive loss of myelin [16, 61]. Indeed, the ALPS index was negatively correlated with the deposition of amyloid-β and tau on PET images [62].

Intriguingly, significant mediation was not observed in all regions that showed significant between-group differences. This explanation can be challenging; however, prior studies involving dynamic imaging and kinetic quantification in rats have demonstrated that glymphatic function exhibits heterogeneous and regionally preferential alterations with aging [63]. Furthermore, a greater enrichment of tracers was observed in poor sleepers than in good sleepers, with the most pronounced differences between poor and good sleepers were observed in the precentral and postcentral cortices and other fronto-temporo-parietal cortices [64]. In this study, a higher percent mediation was also observed in the precentral (40%), supramarginal (33%), postcentral (30%) and pars triangularis (30%) regions. In the WM, a higher percentage mediation was observed in the SLF (42%), which connects the fronto-temporo-parietal lobes, cingulum (38%), which receives vascularization from the anterior cerebral artery, and CST (37%), which originates from neurons in the precentral and postcentral cortices. Our findings may serve as a basis for further studies assessing the association between the enrichment of intrathecally administered contrast agents and myelin content in poor sleepers.

### The associations between myelin differences and the expression of circadian clock genes

Our findings indicated that in GM regions with higher expression of CLOCK, CRY2, PER1, and PER2, notably greater differences in MVF were observed between good and poor sleepers. In contrast, regions exhibiting lower expression of CRY1 were associated with more pronounced MVF differences. It has been suggested that CLOCK, CRY1, CRY2, PER1, and PER2 are expressed in mouse oligodendrocytes and significantly contribute to the proliferation and myelination of OPCs [22, 23]. Furthermore, in a study assessing the impact of sleep deprivation on the expression of circadian clock genes in the cerebral cortex of mice, it was found that CLOCK, CRY2, PER1, and PER2 were upregulated, whereas CRY1 was downregulated following periods of enforced wakefulness [21]. Taken together, the upregulation or downregulation of circadian clock genes because of sleep deprivation may affect the myelin content in the cortex.

### Limitations

This study had some limitations. First, this study used a cross-sectional design and included a relatively small sample size. Future research should incorporate longitudinal data from larger population samples to confirm the temporal correlation between poor sleep-related glymphatic clearance dysfunction and brain tissue demyelination. However, mediation analyses have provided useful insights into this regard. Second, similar to many other studies, we used self-reported measures of sleep quality to categorize participants into good and poor sleepers. Although the PSQI cutoff used in this study exhibited a sensitivity of 89.6% and a specificity of 86.5% in distinguishing between good and poor sleepers [25], future studies may include more objective examinations, such as polysomnography, which is the gold standard for sleep evaluation. Third, MTsat imaging is not specific to myelin as it also measures other tissue macromolecules. However, among all other myelin imaging techniques, MTsat imaging has been shown to exhibit the highest correlation with histopathological myelin content [8]. Fourth, this study did not use MRI-based tracers, which are considered the gold standard for assessing glymphatic function in humans. Furthermore, the ALPS index evaluates perivenous diffusivity only in a limited periventricular region, making it unlikely to accurately represent glymphatic clearance throughout the brain. However, the ALPS index demonstrated a strong correlation with glymphatic clearance function, as evidenced by glymphatic MRI results following the intrathecal administration of gadolinium [18]. Nevertheless, the changes in the ALPS index require careful interpretation. This study mainly focused on the ALPS index; however, several noninvasive alternative methods are available for the evaluation of the human glymphatic system, each with its own advantages and disadvantages [65]. These methods can be employed and compared in further studies. Fifth, the tissue expression of the circadian clock gene was measured postmortem in six adult brains from the AHBA but not in our studied population because data were unavailable. Furthermore, our analyses that link the circadian clock gene expression to the differences in GM MVF were confined to the left hemisphere, constrained by the coverage provided in the AHBA. Future research should extend to the right GM regions. Furthermore, spatial limitations prevented us from correlating the expression of postmortem genes with changes in WM MVF.

## CONCLUSION

Our findings indicate that good sleep quality is crucial for maintaining brain myelin integrity, which in turn preserves cognitive function and reduces depressive symptoms. We also demonstrated that myelin alterations might be mediated by reduced glymphatic clearance and related to regional variations in the expression of circadian clock genes. These insights may be valuable for the development of interventions targeting sleep-related myelin alterations.

## Supplementary Materials

The Supplementary data can be found online at: www.aginganddisease.org/EN/10.14336/AD.2024.0894.

## Data Availability

The data used in this study are available on request from the corresponding author.
