# Supplementary material for: Myelin Changes in Poor Sleepers: Insights into Glymphatic Clearance Function and Regional Circadian Clock Gene Expression
Source: Aging Dis. 2024 Aug 13;16(4):2453–67. doi: 10.14336/AD.2024.0894 (PMC12221392; doi:10.14336/AD.2024.0894)
Supplement: Supplementary file 1 — The Supplementary data can be found online at: www.aginganddisease.org/EN/10.14336/AD.2024.0894. [file AD-16-4-2453-s.pdf]

SUPPLEMENTARY DATA

Myelin Changes in Poor Sleepers: Insights into Glymphatic Clearance Function and Regional Circadian Clock Gene Expression

Christina Andica, Koji Kamagata, Kaito Takabayashi, Zaimire Mahemuti, Manabu Iwasaki, Akifumi Hagiwara, Wataru Uchida, Hiroki Tabata, Hitoshi Naito, Hideyoshi Kaga, Yuki Someya, Yoshifumi Tamura, Ryuzo Kawamori, Hirotaka Watada, Shigeki Aoki

© 2024. Andica C et al. Published online at http://www.aginganddisease.org/EN/10.14336/AD.2024.0894

SUPPLEMENTARY DATA

SUPPLEMENTARY MATERIALS

MRI acquisition

Three-dimensional (3D) T1-weighted images were obtained using a magnetisation-prepared rapid gradient echo protocol (echo time [TE], 2.32 ms; repetition time [TR], 2,300 ms; inversion time, 900 ms; field of view [FOV], 240 × 240 mm; matrix size, 256 × 256; slice thickness, 0.9 mm; and acquisition time, 5.21 min).

Diffusion-weighted images were acquired using echo-planar imaging in the anterior–posterior phase-encoding direction, employing the following parameters: TE, 70 ms; TR, 3,300 ms; FOV, 229 × 229 mm; matrix size, 130 × 130; slice thickness, 1.8 mm; *b*-values, 1,000 and 2,000 s/mm^2; 64 isotropic gradient directions; one non-diffusion-weighted (b = 0) volume; and total acquisition time, 7.29 min. Moreover, standard and reverse phase-encoded blipped images without diffusion weighting (blip up and down) were acquired to correct for magnetic susceptibility-induced distortions associated with echo-planar imaging acquisition [1]. All diffusion-weighted images were thoroughly assessed for artefacts, including gross geometric distortion, signal dropout and bulk motion, across the axial, sagittal and coronal planes. To correct eddy current-induced distortions and subject movements from the diffusion-weighted data, the EDDY tool, integrated into the FMRIB Software Library 5.0.9 (FSL, Oxford Centre for Functional MRI of the Brain, UK; www.fmrib.ox.ac.uk/fsl) [2], was used. To calculate the along the perivascular space index, a fractional anisotropy map for all study participants was generated using the DTIFIT tool, which is a component of FSL. This process relies on the ordinary least squares method [3] and uses diffusion-weighted data with b-values of 0 and 1000 s/mm^2. Diffusivity maps for each subject were then obtained using FSL, particularly in the directions of the x-axis (right–left; Dxx), y-axis (anterior–posterior; Dyy) and z-axis (inferior–superior; Dzz).

Magnetisation transfer saturation (MTsat) imaging used a dual excitation 3D multi-echo fast low-angle shot sequences and was performed with T1, proton density and MT weighting (TE, 1.79/1.79/1.79 ms; TR, 10/24/24 ms; flip angle, 13°/4°/4°; matrix, 128 × 128; slice thickness, 1.8 mm). Parallel imaging was performed with a generalized autocalibrating partially parallel acquisition factor of 2 in the phase-encoding direction; 7/8 partial Fourier acquisition in the partition direction; bandwidth, 260 Hz/pixel; FOV, 224 × 224 mm. For MT-weighted imaging, an off-resonance Gaussian-shaped RF pulse with a frequency offset of 1.2 kHz, duration of 9.984 ms and nominal flip angle of 500° was used. MTsat improves the traditional magnetisation transfer ratio (MTR) by inherently correcting for B1 inhomogeneities and T1 relaxation. This is achieved by approximating the signal amplitude and T1 relaxation at low flip angles with the additional T1-weighted image [4]. Furthermore, MTsat provides superior brain contrast compared with MTR, aligns more closely with quantitative MT measures and is less prone to inconsistencies across different MRI platforms [4-6]. Although the MTsat measure is largely insensitive to B1 inhomogeneities, it still exhibits a residual dependence which introduce bias corresponding to these in homogeneities [7]. To correct for flip angle inaccuracies and further address B1 inhomogeneities in the MTsat maps, we acquired two additional B1 maps using echo-planar imaging with nominal flip angles of 10° and 20° (each acquisition took approximately 10 seconds).

References

[1] Andersson JL, Skare S, Ashburner J (2003). How to correct susceptibility distortions in spin-echo echo-planar images: application to diffusion tensor imaging. Neuroimage, 20:870-888.

[2] Jenkinson M, Beckmann CF, Behrens TE, Woolrich MW, Smith SM (2012). Fsl. Neuroimage, 62:782-790.

[3] Basser PJ, Mattiello J, LeBihan D (1994). Estimation of the effective self-diffusion tensor from the NMR spin echo. J Magn Reson B, 103:247-254.

[4] Helms G, Dathe H, Kallenberg K, Dechent P (2008). High-resolution maps of magnetization transfer with inherent correction for RF inhomogeneity and T1 relaxation obtained from 3D FLASH MRI. Magn Reson Med, 60:1396-1407.

[5] Campbell JSW, Leppert IR, Narayanan S, Boudreau M, Duval T, Cohen-Adad J, *et al.* (2018). Promise and pitfalls of g-ratio estimation with MRI. Neuroimage, 182:80-96.

[6] Karakuzu A, Biswas L, Cohen-Adad J, Stikov N (2022). Vendor-neutral sequences and fully transparent workflows improve inter-vendor reproducibility of quantitative MRI. Magn Reson Med, 88:1212-1228.

[7] Emmenegger TM, David G, Ashtarayeh M, Fritz FJ, Ellerbrock I, Helms G, *et al.* (2021). The Influence of Radio-Frequency Transmit Field Inhomogeneities on the Accuracy of G-ratio Weighted Imaging. Front Neurosci, 15:674719.

© 2024. Andica C et al. Published online at http://www.aginganddisease.org/EN/10.14336/AD.2024.0894

SUPPLEMENTARY DATA

Supplementary Table 1. Comparisons of gray matter MVF between good and poor sleepers.

MVF	Good sleepers		Poor sleepers		*P*-values	FDR-corrected *P*-values
	Mean	SD	Mean	SD		
**Frontal lobe**						
Caudal middle frontal	0.34	0.029	0.32	0.030	0.002	**0.011**
Frontal pole	0.31	0.029	0.29	0.025	0.113	0.153
Lateral orbitofrontal	0.26	0.021	0.25	0.017	0.017	**0.034**
Medial orbitofrontal	0.26	0.021	0.25	0.017	0.002	**0.011**
Pars opercularis	0.26	0.021	0.25	0.024	0.240	0.280
Pars orbitalis	0.29	0.027	0.28	0.027	0.786	0.786
Pars triangularis	0.23	0.019	0.22	0.015	0.018	**0.034**
Precentral	0.25	0.018	0.24	0.018	0.016	**0.034**
Rostral middle frontal	0.30	0.026	0.29	0.022	0.473	0.497
Superior frontal	0.26	0.018	0.25	0.018	0.002	**0.011**
**Temporal lobe**						
Bankssts	0.23	0.020	0.21	0.017	0.004	**0.014**
Entorhinal	0.20	0.012	0.20	0.013	0.213	0.256
Inferior temporal	0.23	0.017	0.22	0.016	0.013	**0.033**
Middle temporal	0.27	0.021	0.26	0.017	0.010	**0.028**
Superior temporal	0.32	0.025	0.30	0.026	0.016	**0.034**
Temporal pole	0.23	0.015	0.22	0.015	0.111	0.153
Transverse temporal	0.24	0.025	0.23	0.023	0.061	0.092
**Parietal lobe**						
Inferior parietal	0.24	0.022	0.22	0.020	0.013	**0.033**
Paracentral	0.32	0.026	0.31	0.031	0.442	0.461
Postcentral	0.25	0.021	0.24	0.023	0.012	**0.033**
Precuneus	0.32	0.020	0.31	0.021	0.008	**0.027**
Superior parietal	0.22	0.032	0.21	0.030	0.184	0.227
Supramarginal	0.25	0.021	0.24	0.019	0.024	**0.044**
**Occipital lobe**						
Cuneus	0.19	0.025	0.18	0.023	0.483	0.509
Fusiform	0.21	0.015	0.20	0.014	0.003	**0.013**
Lateral occipital	0.23	0.025	0.21	0.021	<0.001	**0.010**
Lingual	0.18	0.018	0.17	0.015	0.076	0.081
Pericalcarine	0.097	0.020	0.088	0.018	0.085	0.123
**Limbic regions**						
Caudal anterior cingulate	0.27	0.019	0.25	0.022	0.002	**0.011**
Insula	0.24	0.017	0.23	0.019	0.005	**0.016**
Isthmus cingulate	0.21	0.017	0.20	0.017	0.003	**0.013**
Parahippocampus	0.24	0.018	0.23	0.017	0.054	0.084
Posterior cingulate	0.28	0.020	0.27	0.021	0.001	**0.011**
Rostral anterior cingulate	0.21	0.015	0.20	0.018	0.001	**0.011**
**Deep grey matter**						
Accumbens	0.22	0.013	0.21	0.012	0.157	0.206
Amygdala	0.20	0.009	0.20	0.011	0.428	0.461
Caudate	0.23	0.017	0.22	0.022	0.020	**0.037**
Pallidum	0.24	0.025	0.23	0.026	0.423	0.461
Putamen	0.21	0.011	0.20	0.013	0.004	**0.014**
Hippocampus	0.20	0.013	0.19	0.012	0.024	**0.045**
Thalamus	0.24	0.015	0.23	0.015	0.002	**0.011**
**Cerebellum cortex**	0.18	0.010	0.18	0.008	0.167	0.213

Bold values denote statistical significance (FDR-corrected $P < 0.05$). Abbreviations: Bankssts, banks of the superior temporal sulcus; FDR, false discovery rate; MVF, myelin volume fraction; SD, standard deviation.

© 2024. Andica C et al. Published online at http://www.aginganddisease.org/EN/10.14336/AD.2024.0894

Supplementary Table 2. Comparisons of white matter tract MVF between good and poor sleepers.

MVF	Good sleepers		Poor sleepers		*P*-values	FDR-corrected *P*-values
	Mean	SD	Mean	SD		
ATR	0.39	0.025	0.38	0.026	0.002	**0.004**
CST	0.37	0.021	0.36	0.024	0.038	**0.042**
Cingulum	0.35	0.026	0.34	0.030	0.015	**0.018**
Cingulum hippocampus	0.35	0.022	0.34	0.022	0.010	**0.015**
Forceps major	0.45	0.025	0.43	0.031	0.005	**0.009**
Forceps minor	0.37	0.021	0.35	0.023	<0.001	**0.004**
IFOF	0.37	0.020	0.35	0.023	0.001	**0.004**
ILF	0.40	0.022	0.38	0.027	0.001	**0.004**
SLF	0.31	0.020	0.30	0.022	0.011	**0.015**
UF	0.39	0.025	0.38	0.026	0.002	**0.004**
SLF temporal	0.37	0.022	0.36	0.022	0.070	0.070

Bold values denote statistical significance (FDR-corrected $P < 0.05$). Abbreviations: ATR, anterior thalamic radiation; CST, corticospinal tract; FDR, false discovery rate; IFOF, inferior fronto-occipital fasciculus; ILF, inferior longitudinal fasciculus; MVF, myelin volume fraction; SD, standard deviation; SLF, superior longitudinal fasciculus, UF, uncinate fasciculus.

© 2024. Andica C et al. Published online at http://www.aginganddisease.org/EN/10.14336/AD.2024.0894
